# Functional Blockade of E-Selectin in Tumor-Associated Vessels Enhances Anti-Tumor Effect of Doxorubicin in Breast Cancer

**DOI:** 10.3390/cancers12030725

**Published:** 2020-03-19

**Authors:** Yoshihiro Morita, Macall Leslie, Hiroyasu Kameyama, Ganesh L. R. Lokesh, Norihisa Ichimura, Rachel Davis, Natalie Chakraborty, Nafis Hasan, Roy Zhang, Yuji Kondo, David G. Gorenstein, David E. Volk, Inna Chervoneva, Hallgeir Rui, Takemi Tanaka

**Affiliations:** 1 Stephenson Cancer Center, University of Oklahoma Health Sciences Center, 975 NE, 10th, Oklahoma City, OK 73104, USA; yoshi197@dent.osaka-u.ac.jp (Y.M.); macall-leslie@ouhsc.edu (M.L.); hiroyasu-kameyama@ouhsc.edu (H.K.); norihisa@med.nagoya-u.ac.jp (N.I.); 2 McGovern Medical School, Institute of Molecular Medicine, University of Texas Health Science Center at Houston, 1825 Hermann Pressler, Houston, TX 77030, USA; ganesh@uth.tmc.edu (G.L.R.L.); David.Volk@uth.tmc.edu (D.E.V.); 3 School of Medicine, University of Oklahoma Health Sciences Center, 800 Stanton L. Young Blvd., Oklahoma City, OK 73104, USA; rachel-davis@ouhsc.edu (R.D.); Natalie-Hills@ouhsc.edu (N.C.); 4 Department of Pharmaceutical Sciences, Thomas Jefferson University, 1020 Locust St, Philadelphia, PA 19107, USA; nafis.hasan@tufts.edu; 5 Department of Pathology, College of Medicine, University of Oklahoma Health Sciences Center, 940 SL Young Blvd, Oklahoma City, OK 73104, USA; roy-zhang@ouhsc.edu; 6 Cardiovascular Biology Research Program, Oklahoma Medical Research Foundation, 825 NE. 13th, Oklahoma City, OK 73104, USA; Yuji-Kondo@omrf.org; 7 AM Biotechnologies, LLC, 12521 Gulf Freeway, Houston, TX 77034, USA; david.nmr@earthlink.net; 8 Department of Pharmacology and Experimental Therapeutics, Thomas Jefferson University, 1015 Chestnut St., Philadelphia, PA 19107, USA; Inna.Chervoneva@jefferson.edu; 9 Department of Pathology, Medical College of Wisconsin, 8701 Watertown Plank Rd, Milwaukee, WI 53226, USA; hrui@mcw.edu

**Keywords:** E-selectin, aptamer, doxorubicin, tumor-associated-macrophages

## Abstract

Chemotherapy is a mainstay of treatment for solid tumors. However, little is known about how therapy-induced immune cell infiltration may affect therapy response. We found substantial CD45^+^ immune cell density adjacent to E-selectin expressing inflamed vessels in doxorubicin (DOX)-treated residual human breast tumors. While CD45 level was significantly elevated in DOX-treated wildtype mice, it remained unchanged in DOX-treated tumors from E-selectin null mice. Similarly, intravenous administration of anti-E-selectin aptamer (ESTA) resulted in a significant reduction in CD45^+^ immune cell density in DOX-treated residual tumors, which coincided with a delay in tumor growth and lung metastasis in MMTV-pyMT mice. Additionally, both tumor infiltrating T-lymphocytes and tumor associated-macrophages were skewed towards T_H_2 in DOX-treated residual breast tumors; however, ESTA suppressed these changes. This study suggests that DOX treatment instigates *de novo* intratumoral infiltration of immune cells through E-selectin, and functional blockade of E-selectin may reduce residual tumor burden as well as metastasis through suppression of T_H_2 shift.

## 1. Introduction

Neoadjuvant chemotherapy is indicated for invasive breast cancer treatment as a strategy to improve local control by reducing large, inoperable tumors to a size amenable to surgical resection [1] and to gain systemic control over micrometastases [2,3]. While over 80% of breast tumors clinically respond to combination chemotherapy with a measurable size reduction [3], the likelihood of complete tumor eradication (complete pathologic response) is relatively low and residual tumor remains in 70–85% of cases [4,5]. Studies suggest a possible predictive role of pre-treated tumor stroma composition in response to neoadjuvant chemotherapy. In chemotherapy-naïve breast tumors the prevalence of tumor associated macrophages (TAMs) as well as T-lymphocytes (TIL) is inversely associated with chemotherapy response [6,7]. Similarly, studies reported a predictive role of *de novo* infiltration of immune cells into tumors on the outcome. Clinical studies showed that increased immune cell infiltration during chemotherapy is correlated with higher residual tumor burden and adverse clinical outcomes [8,9]. Preclinical studies also indicated an increase in immune cell density in chemotherapy-treated residual breast tumors, and pharmacologic inhibition of immune cell infiltration improved therapeutic efficacy [6,7,8,9,10,11]. These studies suggest possible negative impacts of therapy-related *de novo* immune cell infiltration on therapeutic efficacy.

Doxorubicin (DOX) is indicated for treatment of invasive breast cancer as a part of combination chemotherapy. DOX causes cytotoxic death of cycling cells [6], which instigate successive infiltration of immune cells [12]. Infiltration of immune cells into damaged tissue from the circulation is governed by the adhesion cascade, where circulating cells adhere to and transmigrate through inflamed vessels under swift blood flow [13,14,15]. E-selectin (CD62E, ELAM-1, LECAM-2) expression on vascular surfaces is a hallmark of inflammation that mediates capture of circulating immune cells on the vessel surface through affinity binding with its counter ligands (sLex, sLeA, CD44 (HCELL), and PSGL) [16]. E-selectin expression is spatiotemporally limited and induced in response to inflammatory cytokines [17]. Accordingly, E-selectin expression in tumor-associated vessels is elevated in different types of carcinomas [18,19,20], and the abundance of sLex positive immune infiltrates in the tumor is inversely associated with prognosis [17]. Therefore, we hypothesized that functional blockade of E-selectin reduces chemotherapy-associated *de novo* infiltration of immune cells by obstructing their entry to the tumor, in turn, mitigating residual tumor burden.

## 2. Results

### 2.1. High CD45^+^ Immune Cell Density in Chemotherapy-Treated Residual Breast Tumors

**Figure 1:**
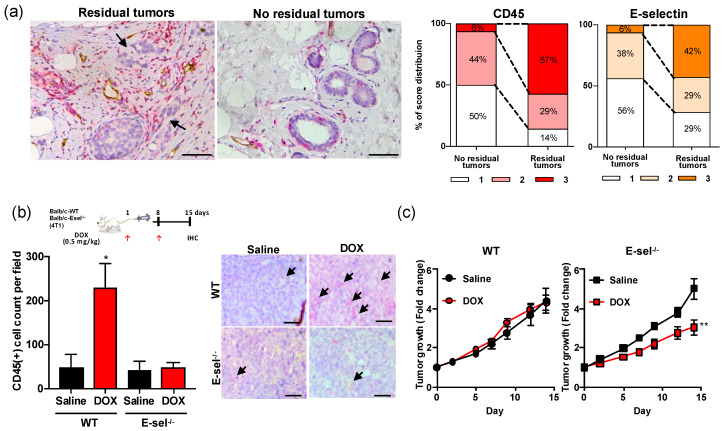
High CD45^+^ density in DOX-treated breast tumors. (**a**) CD45^+^ immune cells and E-selectin^+^ inflamed vessels in human breast tumors that were treated with DOX containing neoadjuvant chemotherapy. Representative images of double immunohistochemistry show CD45 (red), E-selectin (brown), and hematoxylin counterstaining (blue) in cases of residual and no residual tumor. The scoring index of CD45 and E-selectin were scored as 1 (minimal), 2 (moderate), or 3 (abundant). Score distribution was summarized as %. Scale bar indicates 100 μm. (**b**) Immunohistochemistry of CD45 of DOX-treated tumors in the WT and E-selectin^−/−^ mice. 4T1 tumor-bearing mice (*n* = 5) were treated with saline or 0.5 mg/kg DOX (DOX0.5). CD45^+^ cells were counted from 3 representative images of 5 individual tumors and summarized as % of CD45^+^ cells per field of view at a final magnification of ×200. Scale bar indicates 100 μm. Graph is depicted as mean ± SE. Statistical significance was analyzed by the Mann Whitney test. (**c**) Tumor growth rate of DOX-treated E-selectin^−/−^ and WT mice bearing 4T1 tumors. *, *p* < 0.05; **, *p* < 0.01.

To assess the association between immune cell density and therapy response, we histopathologically quantified CD45^+^ immune cells and E-selectin^+^ inflamed vessel density in surgically resected invasive human breast tumors (Stage II–III), treated with DOX-containing neoadjuvant chemotherapy (Supplementary Figures S1 and S2). CD45^+^ cells were densely present in 57% cases with residual tumor, yet only noted in 6% cases with no residual tumor (Figure 1a). Vessels adjacent to the residual tumor were inflamed as characterized by elevated expression of E-selectin (Figure 1a). Inflammation score, defined by the abundance of CD45^+^ immune cell clusters and E-selectin^+^ vessels, was disproportionally high around residual human breast tumors (score of 3 in 42% of residual vs. 6% of no residual tumor cases; Figure 1a). The spatial proximity of CD45^+^ immune cell clusters and E-selectin^+^ vessels suggested a possible role of E-selectin as the gateway for circulating immune cells. E-selectin null (E-selectin^−/−^) or wildtype (WT) mice bearing 4T1 murine breast tumors were treated once weekly with intravenous injection of saline or low dose DOX (0.5 mg/kg). DOX treatment resulted in a 4.6-fold increase of CD45^+^ immune cell density compared to the saline control group in WT mice (Figure 1b), whereas the CD45^+^ density remained unchanged (1.1 fold over saline control) in E-selectin^−/−^ mice. Although once a week treatment with low dose DOX showed no significant effect on tumor growth in WT mice (7.1% growth reduction; Figure 1c), the tumor growth rate of DOX-treated E-selectin^−/−^ mice was markedly augmented (39.8%) compared to saline controls (Figure 1c). No significant weight loss was noted among any treatment group (Supplementary Figure S3). Together, this data suggests a new therapeutic opportunity by blocking DOX treatment-related immune cell infiltration via E-selectin for the improvement of anti-tumor therapy.

### 2.2. Binding of E-Selectin Aptamer to Inflamed Tumor Vessels

**Figure 2:**
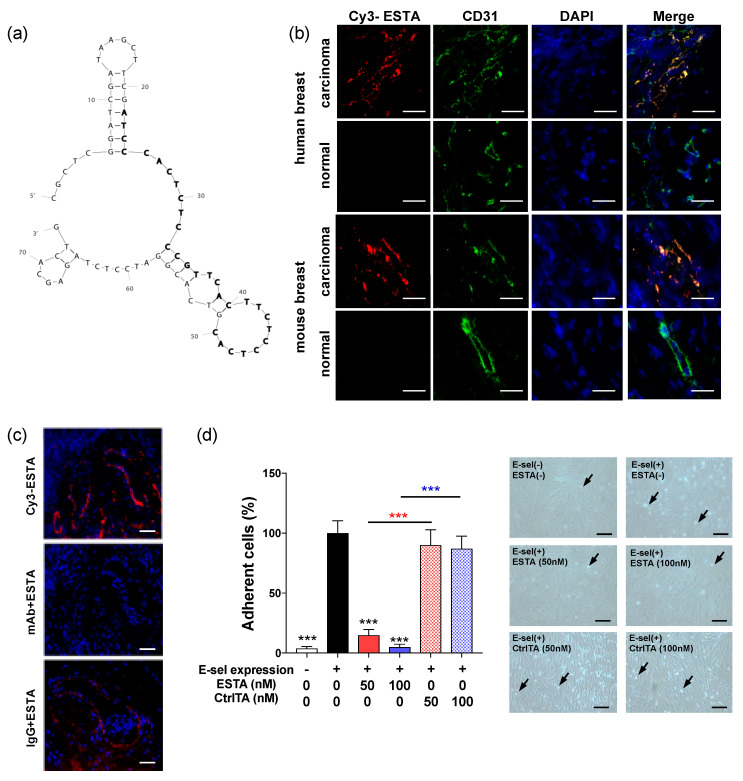
Binding of ESTA to E-selectin expressing vessels in the tumor. (**a**) M-fold structure of ESTA. (**b**) ESTA binding to the vessels in human and mouse breast tumors. Frozen section of human breast tumors (*n* = 3) and normal breast tissues (*n* = 2), mouse breast tumors derived from MMTV-PyMT (*n* = 6), and normal mammary fat pad from non-carrier control female mice (*n* = 6) were incubated with 50 nM Cy3-labeled ESTA (red) and then immunostained with CD31 (green). The nucleus was counterstained with DAPI (blue). Images were captured in the same condition at a final magnification of ×400. Scale bar indicates 10 μm. (**c**) ESTA binding to E-selectin expressing vessels in human breast tumors. Serial frozen sections of human breast tumors were incubated with either saline, E-selectin monoclonal antibody, or IgG control before incubation with 50 nM Cy3-labeled ESTA (red) and counterstain with DAPI (blue). Representative images were taken at a final magnification of ×200. The scale bar indicates 50 μm. (**d**) Flow adhesion assay of PBMCs to E-selectin expressing ES-HMVECs. Following induction of E-selectin expression by doxycycline, the cells were treated with either saline, ESTA, or CtrlTA (50 or 100 nM) for 1 h. A pool of human PBMCs (1.5 × 10^6^ cells/mL) was infused into a flow chamber for 3 min at 1 dyn/cm^2^. Arrows in representative images indicate PBMCs that adhered to ES-HMVECs at 100× magnification. Data from the doxycycline-incubated, untreated group was shown as 100%. The data represent mean ± SD from at least a duplicate of three independent experiments, and statistical significance was determined by the Mann Whitney test. ***, *p* < 0.001. The scale bar indicates 50 μm.

An anti-E-selectin aptamer (ESTA) was developed for functional blockade of E-selectin (Figure 2a) [21]. Incubation of Cy3-labeled ESTA (50 nM) with breast tumors derived from humans and MMTV-PyMT transgenic mice displayed intense, localized binding (red fluorescence) to vessels that co-localized with CD31 (green fluorescence) as shown in yellow overlay (Figure 2b). In contrast, Cy3-labeled ESTA binding to the vessels was absent in respective normal counterparts in both mice and humans. Similarly, Cy3-ESTA showed no notable binding to vessels in other healthy organs (data not shown). Additionally, neutralization of E-selectin with monoclonal antibody completely abolished subsequent Cy3-labeled ESTA binding to tumor vessels in serial sections from a human breast tumor (Figure 2c). Normal IgG control did not affect subsequent Cy3-labeled ESTA binding to the vessels, providing evidence of E-selectin specific binding in both mouse and human breast carcinomas without non-specific binding to normal vessels. To further determine the ability of ESTA to inhibit E-selectin function, the shear resistant adhesion assay was performed using human peripheral blood mononuclear cells (PBMCs) to E-selectin inducible tet-on human microvascular endothelial cells (ES-HMVECs) under physiologic flow. Induction of E-selectin expression by doxycycline resulted in a 25.8-fold increase in shear resistant adhesion of PBMCs to ES-HMVECs as shown by arrows, compared to baseline without induced E-selectin expression (Figure 2d). However, ESTA (100 nM) completely abrogated E-selectin-dependent shear resistant adhesion. The adhesion of PBMCs was unaffected by the control aptamer (CtrlTA). ESTA showed no effect on the viability of PBMCs, Jurkat cells, HL60 cells (Supplementary Figure S4), and HMVECs [21]. These data support the ability of ESTA for specific binding to E-selectin expressing vessels and blockade of immune cell adhesion onto the endothelial surface.

### 2.3. ESTA Enhanced the Anti-Tumor Effect of DOX

**Figure 3:**
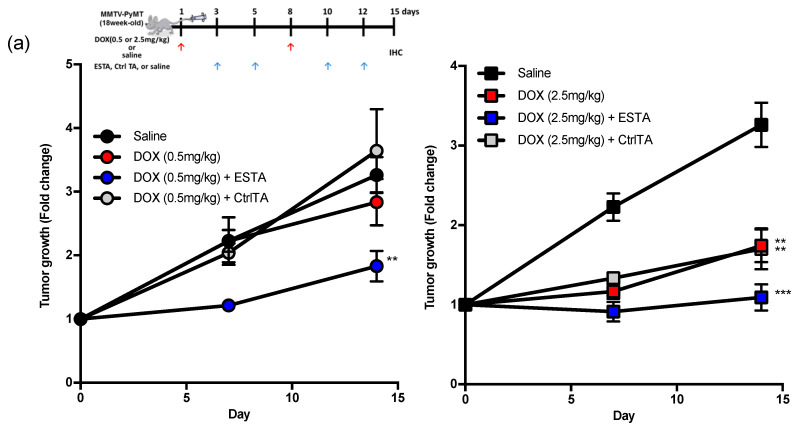

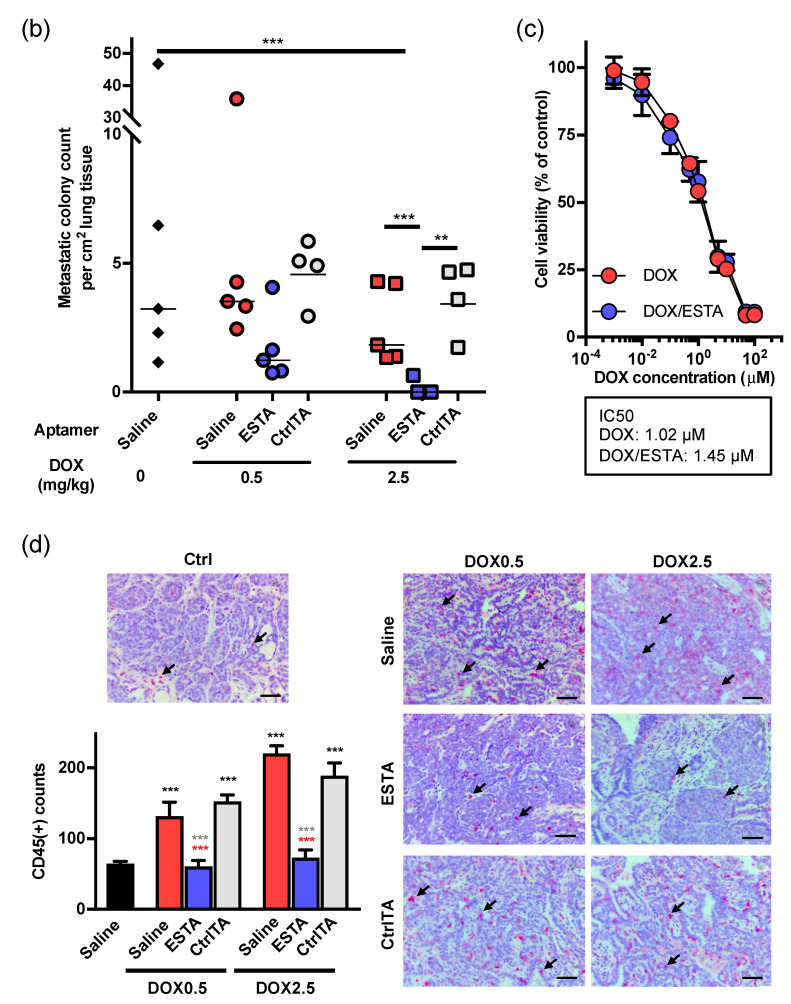
The effect of ESTA on DOX therapy. (**a**) Tumor growth in MMTV-PyMT mice. Mice (*n* = 6) were treated with DOX (0.5 or 2.5 mg/kg), DOX/ESTA, or DOX/CtrlTA for 2 weeks as depicted in the timeline. The graph shows fold change of tumor growth (0 days/treatment starting point as 1 of each group at days 0, 7, and 14. (**b**) The effect of ESTA/DOX combination on lung metastases. Step sections of the whole lung were stained with H&E and metastatic foci sizes of >50 μm ϕ were counted and normalized by total area (cm^2^). (**c**) The effect of ESTA on IC50 of DOX. Py8119 cells were incubated with various concentration of DOX (0.001, 0.01, 0.1, 1, 10, 100 μM) with saline or ESTA (100 nM) for 24 h. Cell viability was measured by MTT assay (*n* = 6). The viability of saline-treated cells was 100%. IC_50_ was calculated by four parameter logistic curves. (**d**) Immunohistochemistry of CD45. Tumors were stained with CD45 (red) and counterstained with hematoxylin (blue). Arrows indicate CD45^+^ cells. The bar graph depicts mean ± SE, and statistical significance was analyzed by the Mann Whitney test. Scale bar indicates 50 μm. Graph is depicted as mean ± SE (a, b) or ± SD (d). Statistical significance was analyzed by the Mann Whitney test. *, *p* < 0.05; **, *p* < 0.01; ***, *p* < 0.001.

We next examined whether ESTA improves the anti-tumor effect of DOX. Female MMTV-PyMT mice were treated once weekly with two doses of DOX (high, 2.5 mg/kg; low, 0.5mg/kg), followed by twice weekly with either ESTA or CtrlTA once the tumor reached 200 mm^3^ as shown in the treatment scheme. Two weeks treatment with high dose DOX significantly delayed tumor growth, whereas low dose DOX treatment showed no significant anti-tumor effect (Figure 3a). A combination of ESTA with low dose DOX significantly reduced the tumor growth rate compared to the same dose DOX alone (Figure 3a and Supplementary Figure S5a,b). High dose DOX/ESTA combination demonstrated sustainable tumor growth inhibition over 2 weeks compared to DOX alone. The tumor volume of low dose DOX/ESTA treated mice was similar to that of high dose DOX-treated ones (Supplementary Figure S5b), accounting for an approximately 5-fold dose reduction. ESTA alone, however, showed no effect on tumor growth (Supplementary Figure S5d). These studies suggested that ESTA intensifies the anti-tumor effect of DOX, but by itself has no effect on tumor growth in mice. Additionally, a lack of effect of CtrlTA indicates that the effect of ESTA is not due to a non-specific effect of the nucleic acid on the immune response (Figure 3a). The high dose DOX/ESTA combination significantly reduced lung metastases compared to DOX alone (1.83 vs. 0.13 metastatic colonies per cm^2^; Figure 3b). No obvious weight loss was noted in any treatment group in MMTV-PyMT mice (Supplementary Figure S5c). Notably, the IC_50_ of DOX was unchanged by the addition of ESTA (1.02 μM in DOX alone vs. 1.45 μM in DOX/ESTA; Figure 3c); thus, a synergy between DOX and ESTA on tumor growth inhibition was not due to the enhancement of DOX cytotoxicity, suggesting, rather, indirect effects. The density of CD45^+^ immune cells in the tumor stroma, excluding necrotic areas and peripheral stroma, was 2.0- and 3.4-fold higher in low and high dose DOX-treated turmors, respectively, compared to tumors in the saline control group (Figure 3d). However, CD45^+^ immune cell density was far less in DOX/ESTA-treated tumors than those treated with the same dose of DOX in MMTV-PyMT mice (Figure 3d), suggesting that ESTA may mitigate DOX-induced CD45^+^ immune cell infiltration. 

### 2.4. ESTA Suppressed DOX Treatment-Associated T_H_2 Shift

**Figure 4:**
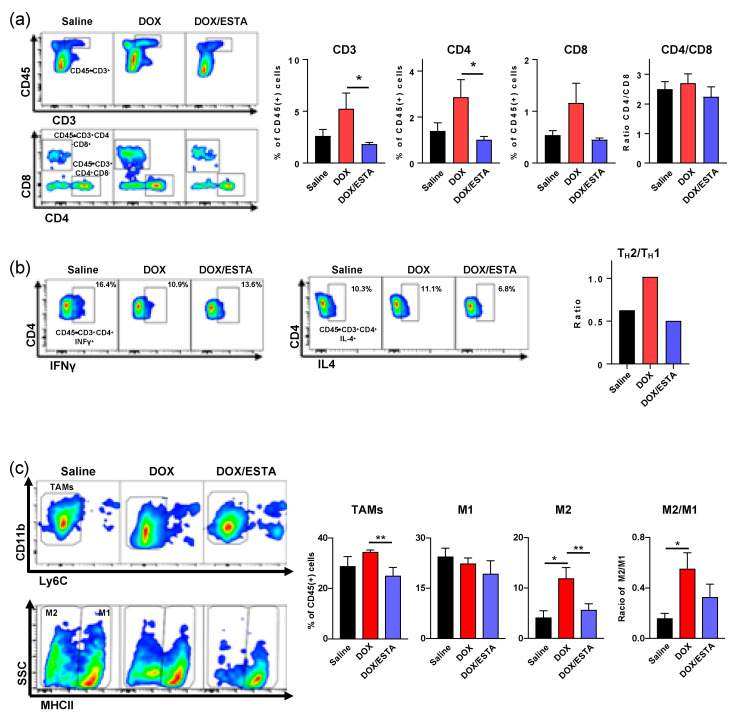

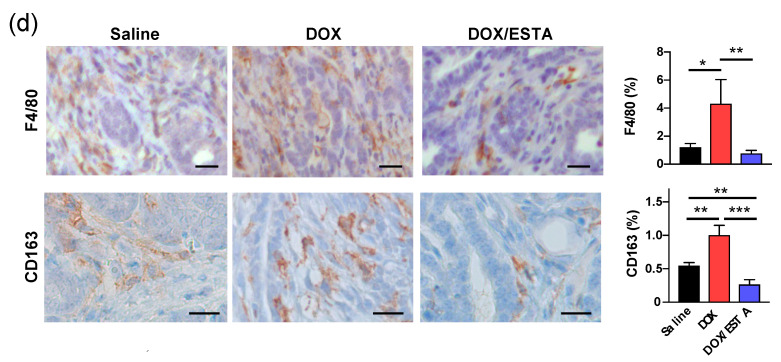
Suppression of immune cell infiltration by ESTA in DOX-treated tumors. (**a**) FACS analysis of TILs in tumors derived from py8119 cells. The data were depicted as mean ± SE and analyzed by the Student’s *T*-test. (**b**) Analysis of TIL subsets. A pool of tumors (*n* = 6) analyzed by FACS for T_H_1 and T_H_2, following activation. (**c**) FACS analysis of TAMs in breast tumors of MMTV-pyMT mice. The data are depicted as mean ± SE and analyzed by the Student’s T-test. *, *p* < 0.05; **, *p* < 0.01. (**d**) Immunohistochemistry of TAMs. Representative images show overall TAMs (F4/80) and M2 TAMs (CD163) in brown and the nucleus in blue. Graphs summarize TAMs and M2 TAMs in stromal area (cells / stromal area mm^2^) at a final magnification of ×200. Scale bar indicates 20 μm. The cells were counted from three representative images of six individual tumors. Statistical significance was analyzed by the Student’s T-test. *, *p* < 0.05; **, *p* < 0.01; ***, *p* < 0.001.

Since the enhanced anti-tumor effect of the DOX/ESTA combination was accompanied by a profound decrease in intratumoral CD45^+^ density, we next performed multicolor flow cytometry (FACS) to identify the affected immune cell subsets (Supplementary Figure S6). Weekly DOX treatment resulted in an approximately 2-fold increase of CD3^+^, CD4^+^, and CD8^+^ T-lymphocytes in py8119 tumors (Figure 4a). However, DOX/ESTA treatment resulted in a reduction of T-lymphocyte proportions to a level similar to saline control (Figure 4a). While proportion of CD3^+^, CD4^+^, and CD8^+^ T-lymphocytes increased with DOX treatment, the CD4/CD8 ratio was unaffected among treatment groups. Furthermore, T-lymphocytes subset analysis of a pool of tumors showed that DOX treatment results in a T_H_2 shift, which was suppressed by ESTA (Figure 4b). Further analysis of intratumoral macrophage subsets showed that DOX treatment resulted in an increase of M2 TAM proportion (CD45^+^ F4/80+ CD11b+ Ly6Clow Ly6Glow MHC II^low^) with no significant changes in M1 TAM (CD45^+^ F4/80+ CD11b+ Ly6Clow Ly6Glow MHC II^hi^) in MMTV-pyMT mice, skewing tumors towards the M2 state, while ESTA suppressed this change (Figure 4c). Consistently, histopathologic analysis supported a 2.1- and 1.8-fold increase in F4/80^+^ TAMs and CD163^+^ M2 TAMs in DOX-treated mouse tumors, respectively, compared to saline controls (Figure 4d). However, ESTA suppressed this change, to a level slightly lower than that in saline controls. Based on the profound changes noted in CD45^+^ density in histologic analysis (Figure 3), the extent of proportional changes in immune subsets shown in FACS analysis was presumably attenuated as the data was normalized by CD45.

### 2.5. ESTA Suppressed T_H_2-Related Pro-Tumorgenic Changes

**Figure 5:**
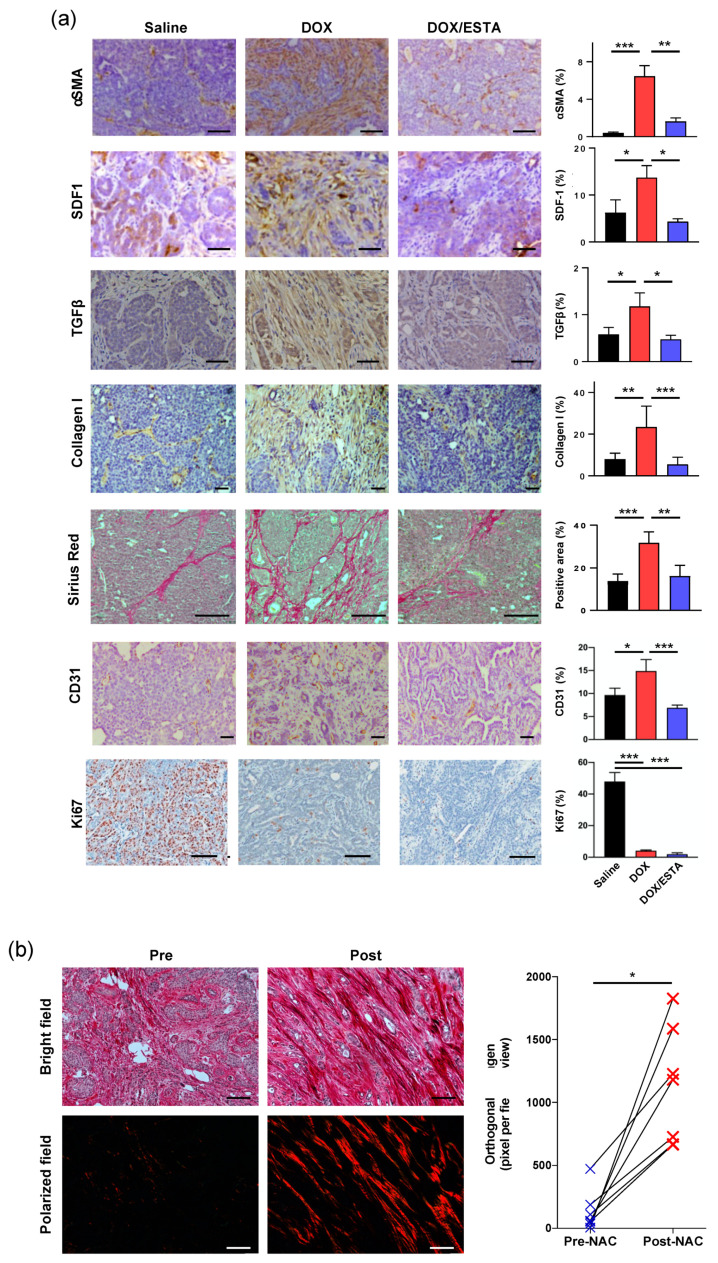
ESTA suppressed DOX-induced pro-tumorgenic changes. (**a**) Immunohistochemical analysis of tumor stroma components. MMTV-PyMT breast tumors (*n* = 6) treated with saline, DOX, or DOX/ESTA were analyzed. Five representative images were taken from each mouse and quantified as % of stromal area (mm^2^). Scale bar indicates 50 μm. The data were summarized in the graph as mean ± SE. Statistical significance was analyzed by Student’s T-test. *, *p* < 0.05; **, *p* < 0.01; ***, *p* < 0.001. (**b**) Elevated collagen deposition in DOX treated surgically resected residual human breast tumors (post-treatment) and their matched biopsy tumors (pre-treatment) were stained with Picro-Sirius Red and counterstained with Weigert’s hematoxylin (*n* = 7). Representative images captured by bright-field (parallel) and polarized light (orthogonal collagen) are shown. Orthogonal collagen was measured as the percentage of total unmasked pixels except perivascular in the field of view at a final magnification of ×100. Scale bar indicates 100 μm. The graph depicted individual cases of orthogonal collagen pre- and post-treatment. Data were analyzed by the Kruskal–Wallis test, *, *p* < 0.05, Cl = 95%.

**Figure 6:**
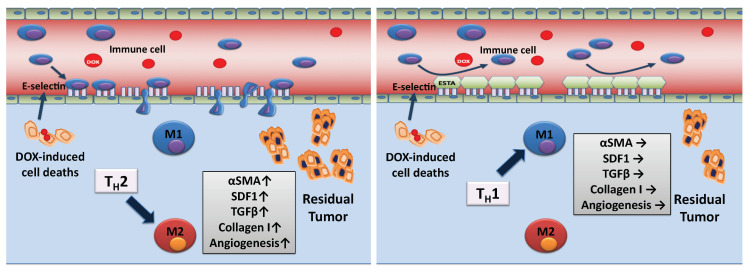
Schematic working model of ESTA’s mechanism of action. DOX-induced cell death provokes infiltration of immune cells through E-selectin expressing inflamed vessels, skewing the tumor towards T_H_2. T_H_2 cytokines induce pro-tumorigenic (proliferation and angiogenesis) and pro-fibrotic changes. The binding of ESTA to E-selectin expressing vessels obstructs the adhesion of immune cells to the luminal surface of vessels, subsequently suppressing DOX treatment-induced pro-tumorigenic and pro-fibrotic changes.

Given the suppression of the DOX-associated T_H_2 shift by ESTA, histopathologic analyses were performed using MMTV-pyMT tumors to evaluate T_H_2-releated pro-tumorigenic changes [22]. Significantly increased expression in α-SMA (16 fold), SDF-1 (2.1 fold), TGFβ (2.0 fold), and CD31 (1.5 fold) were noted in DOX-treated tumors compared to saline control (Figure 5a). However, ESTA suppressed DOX-associated pro-fibrotic and pro-angiogenic changes. DOX treatment resulted in a reduction in Ki67 (11 fold) regardless of the presence of ESTA, compared to saline control. To further validate DOX treatment-associated fibrotic changes, quantitative and qualitative changes in interstitial collagen were determined using surgically resected invasive human breast tumors that were treated with DOX containing chemotherapy and their matched biopsy tumors (Stage II–III). Bright field images of pre-treatment tumors showed extensive collagen fibrils randomly dispersed in between cancer cell clusters. In contrast, residual breast tumors showed more abundant collagen fibers bordering cancer cell clusters (Figure 5b). Polarized images confirmed a marked increase in collagen linearity, length, and thickness in residual human breast tumors. Semi-quantitative analysis revealed a steep increase in orthogonal collagen among residual tumors (i.e, post-treated) compared to their matched biopsy tumors (pre-treated; 0.6 vs. 11.8). In conclusion, our study demonstrated that suppression of DOX-associated *de novo* infiltration of immune cells through E-selectin functional blockade mitigates T_H_2 shift, pro-tumorigenic, and pro-fibrotic changes (Figure 6).

## 3. Discussion

Anthracyclines (e.g., doxorubicin: DOX) are commonly integrated into first-line chemotherapy regimens and indicated for many types of cancer, including breast cancer. Cell death instigates successive and sequential recruitment of a large number of circulating immune cells shortly after DOX administration [12]. Accordingly, increased immune cell infiltration and composition changes have been detected in chemotherapy-treated residual breast tumors in both mice and humans [6,10,11]. Our study showed a substantial increase of CD45^+^ cells in DOX-treated residual mouse and human breast tumors. A recent study indicated that abundant TILs and TAMs in post-chemotherapy residual breast tumors are associated with shorter disease-free progression time [8], suggesting a possible inverse association between therapy-induced immune cell infiltration and therapeutic response. Functional blockade of E-selection, a gateway for infiltrating immune cells into damaged tissues, using genetic ablation and ESTA markedly reduced tumor growth rate when combined with DOX. ESTA was neither cytotoxic by itself nor an enhancer of DOX cytotoxicity, rather, administration of ESTA allowed an approximately five-fold reduction in dose of DOX to achieve equal therapeutic effect perhaps through the indirect effect of suppressing the DOX treatment-associated T_H_2 shift. Anthracycline-induced cardiomyopathy, including oxidative stress [23,24,25], IL-1-mediated inflammation [23,26,27,28], and cardiac apoptosis [23,24], limits the allowable cumulative lifetime dose; thus, reduction of DOX dosage while maintaining efficacy is an attractive therapeutic strategy. ESTA blocked de novo infiltration of CD45^+^ cells and the T_H_2 shift in the DOX-treated tumors; however, the effect of DOX/ESTA on other ogans remains unaddressed. The outcomes of T_H_1 dominance in the tumor vs. other organs, particularly the heart, are expected to be different, given the role of IL-1 in DOX-associated cardiac toxicty [29]. Therefore, further comprehensive analyses including immunoprofiles and cytokine panel in other organs necesitate a full evaluation to understand the benefit of ESTA therapy.

Theoretically, blockade of tissue infiltration of immune cells is attainable through targeting a key mediator in the adhesion cascade (i.e., immune cells, cytokines/chemokines, or vessels). Thus far, immune cells (α4 integrin [30] or CD11b/CD18 [31]) and chemokines (macrophage colony stimulating factor receptor (CSF1R [32]) have been successfully targeted by monoclonal antibodies for impediment of immune cell infiltration into tumors or other tissues. These antibodies, in combination with chemotherapy or radiation, enhanced the therapeutic response in murine models of breast cancer [6,31]. Consistent with findings from the previously mentioned studies, targeting the adhesion cascade via E-selectin expressing vessels using ESTA provides additional evidence for the validity of anti-migration strategy in conjunction with DOX. Implementation of an anti-migration therapy, however, may require careful considerations of the potential for compensatory influx of other immune cell subsets into the tumor [33], impairment of immunosurveillance, infection, delay of wound repair, and so on. Thus, it is critical to establish a stratgegy for patient stratification and treatment scheduling (e.g., duration and administration sequence with chemotherapy). Given the pivotal role of E-selectin as a mediator of tissue migration and its spatiotemporally limited expression on the luminal surface of inflamed vessels, different chemical entities of antagonistic ligands have been developed [21,34,35,36,37,38].

Advantages of aptamers over antibodies or small molecule inhibitors may include low or no immunogenicity [39,40], high tolerability [41], low production cost, and long shelf-life [42], while nuclease sensitivity [43,44], rapid renal clearance [44,45], and interactions with serum components [46] collectively contribute to a common shortcoming of aptamers, their short serum half-life. In this line, daily intravenous administration of ESTA was necessary to achieve the tumor growth inhibition due to the rapid clearance of unmodified ESTA, although this dosing schedule was well tolerated in mice [47]. The biodistribution of an intravenously injected aptamer largely differs by target, and tissue uptake of the aptamer in normal organs except for kidneys and liver, is generally low [48]. The improvement of pharmacokinetic and pharmacodynamic properties is essential for successful clinical translation of aptamer therapeutics.

In summary, our study showed a reduction of immune cell influx through blockade of E-selectin readily suppresses DOX-induced T_H_2 shift and fibrosis, highlighting the potential advantage of controlling DOX-induced immune cell infiltration and presenting an opportunity for supportive care in combination with DOX that could potentially improve outcomes and reduce adverse effects in BC treatment.

## 4. Materials and Methods 

### 4.1. Clinical Samples

Stage II–III breast cancer patients were consented to a study approved by the IRB committee of the University of Oklahoma Health Sciences Center from 2014–2016. Formalin-fixed paraffin-embedded (FFPE) whole mount tissue sections of surgically resected tumors following DOX-containing neoadjuvant chemotherapy (AC-T) were used for immunohistochemistry. PBMCs were collected from the whole blood of healthy donors (*n* = 3). Frozen sections of breast tumors (*n* = 3) were retrieved from the tissue archive at Thomas Jefferson University from 2011–2012. Frozen sections of normal tissues were purchased from US Biomax (Derwood, MD, USA).

### 4.2. Cell Culture

Murine breast cancer cell lines, 4T1 and py8119 (ATCC, Rockville, MD) were cultured in Dulbecco’s modified Eagle medium (Corning, Corning, NY, USA) and RPMI, respectively, supplemented with 10% fetal bovine serum (FBS), 1% glutamax, and 1% antibiotic-antimycotic (Thermo Fisher Scientific, Waltham, MA, USA). MITO extender was added to py8119 cells (BD Bioscience, San Jose, CA, USA). E-selectin inducible tet-on human microvascular endothelial cells (ES-HMVEC) were cultured in endothelial basal medium-2 (EBM-2) (Lonza, Basel, Switzerland) supplemented with 2% tet-approved FBS (Clontech Laboratories, Mountain View, CA, USA) and EGM SingleQuot Kit containing epidermal growth factor (EGF), hydrocortisone, and GA-1000 (Lonza). E-selectin expression was induced with 1000 µg/mL of doxycycline for 5 h as described previously [49]. All cells were cultured in 5% CO_2_ humidity chambers at 37 °C.

### 4.3. Aptamer Binding to Tumor Vasculature

ESTA (5′-CGCTCGGA*TCGA*TA*A*GCTTCGA*TCCCA*CTCTCCCGTTCA*CTTCTCCTCA *CGTCA*CGGA*TCCTCTA*GA*GCA*CTG-3′) and CtrlTA (5′-CCCACTTA*TCGTCCCTTAA *TGA*GTTTA*CTCGCA*CACCGGACAGCCGTCGGATGGCTGGATCCG*TAGCGGTCCGG-3′) were synthesized chemically and purified as described previously (asterisks indicate adenines with monothiophosphate substitution) [21,50]. The tissue sections were fixed with 4% paraformaldehyde and incubated with 50 nM Cy3-labeled ESTA, then immunostained with anti-CD31 antibody and counterstained with DAPI. For antibody blocking, serial sections were pre-incubated with E-selectin antibody or IgG control before incubation with Cy3-labeled ESTA.

### 4.4. Immunohistochemistry

Histopathologic analysis was performed on FFPE sections (4 μm). Double immunohistochemistry for E-selectin and CD45 in human breast carcinoma was performed as previously described [51]. The background was corrected through immunohistochemistry by replacing antibody with IgG. Slides were evaluated by a board-certified pathologist (RZ). E-selectin vascular expression was graded as 1 (none to weak), 2 (modest), or 3 (high). Immunohistochemical staining of CD45 was quantitatively categorized as a score of 1 (≤30 cells), 2 (31 to 100), or 3 (>100) CD45^+^ cells around residual tumors containing invasive cells in the field of view, respectively. Metastatic foci present in mouse lung were counted from all lobes and normalized by tissue area. All images were captured with a Leica DM2500 microscope connected to a Leica DFC425C camera (Leica, Wetzlar, Germany). All slides were scanned and quantified using Aperio (Leica). For immunohistochemistry of mouse tumors, after deparaffinization and rehydration, antigen retrieval was performed using Envision Flex Target Retrieval Solution (pH 6.1, Dako, Santa Clara, CA, USA) in a pressure cooker for 20 min at 110 °C. Endogenous peroxidase and nonspecific epitopes were blocked with 0.3% hydrogen peroxide. Sections were incubated with the primary antibody followed by the corresponding secondary antibody as listed in Supplementary Figure S7. Expression of CD45 and other markers was visualized using aminoethylcarbazole and 3,3-diaminobenzidine, respectively, followed by counterstain with CAT Hematoxylin (Biocare Medical, Pacheco, CA, USA). The number of positive cells in five randomly selected high-power fields exclusive of necrotic areas and non-peripheral stroma at ×200 magnification were counted and normalized by field of view of area of interest (stroma or cancer cells).

### 4.5. Adhesion Assay

ES-HMVECs were grown to confluence in a flow chamber (µ-Slide I 0.4 Luer; Ibidi, Madison, WI, USA) coated with collagen and fibronectin as described previously [49]. After incubation with 1000 ng/mL doxycycline for 5 h, a pool of human PBMCs (5 × 10^5^ /mL) was infused into a flow chamber at one dyn/cm^2^ shear stress for 5 min.

### 4.6. Mouse Models

Mice were obtained from the Jackson Laboratory (Bar Harbor, ME, USA) and maintained in a pathogen-free facility. For syngeneic grafting tumor models, 4T1 cells were injected into the abdominal mammary fat pads of 6 week-old female E-selectin^−/−^ or WT Balb/c mice. Py8119 cells were injected into the mammary fat pad of C57BL/6J as the cells were originated from B6.FVB-Tg (MMTV-PyVT) 634Mul/LellJ. Treatment was initiated when tumor volume reached approximately 100 mm^3^ as indicated in the schedule timeline. B6.FVB/MMTV-PyMT (MMTV-PyMT) were bred, and females were maintained until tumor size reached 250 mm^3^ on either thoracic or abdominal fat pad. All mice were intravenously administered saline, DOX, or aptamer via the tail vein. Control mice received equivolume of saline for equal manipulation frequency. Tumor size was measured by caliper, and volume was calculated using the formula, L × W^2 × 0.52, where L is the long diameter, and W is the short perpendicular diameter.

### 4.7. Preparation of Intratumoral Immune Cells and Flow Cytometry

A single cell suspension from mouse tumors was prepared and subjected to FACS analysis [52]. Briefly, mouse tumors were cut into small pieces, treated with collagenase A, and filtered through a 70 µm strainer. Leukocytes were isolated by using Fico/Lite LymphoH density gradient sedimentation (Atlanta Biologicals, Lawrenceville, GA, USA). Cells were incubated with Zombie Aqua viability kit (Biolegend, San Diego, CA, USA) to stain dead cells and Fc-block (Biolegend) to prevent nonspecific binding. Cell membrane antigens were stained with antibodies described in Supplementary Figure S7. The cells were analyzed using a BD LSRII analyzer. Data were acquired using BD FACSCanto II (BD Biosciences, San Jose, CA, USA) and FlowJo software version 10 (Tree Star, Ashland, OR, USA).

### 4.8. Statistical Analysis

Data obtained from in vitro experiments were statistically analyzed to provide 95% power for test at a significance level of 0.05. The results are presented as mean ± SD for in vitro and mean ± SEM for in vivo experiments, respectively. Comparisons between two groups were analyzed using Student’s t-test (*in vitro*) or Mann Whitney test (*in vivo*) by Prism 7 software (GraphPad Software, La Jolla, CA, USA).

## 5. Conclusions

This study suggests that DOX treatment instigates de novo intratumoral infiltration of immune cells through E-selectin, and functional blockade of E-selectin may reduce residual tumor burden as well as metastasis through suppression of T_H_2 shift. Anti-migration therapy to mitigate therapy-induced immune cell infiltration together with chemotherapy may present a promising strategy for improved outcomes in breast cancer treatment.

## Supplementary Materials

The following are available online at https://www.mdpi.com/2072-6694/12/3/725/s1, Figure S1: Scoring index of CD45 and E-selectin., Figure S2: Demographic and clinical characteristics of DOX-containing neoadjuvant chemotherapy-treated (AC-T regimen) human breast carcinoma cases, Figure S3: Body weight measurement of mice assigned to Figure 1c study, Figure S4: The effect of ESTA on cell viability, Figure S5: Tumor growth and body weight measurements of MMTV-PyMT mice, Figure S6: Gates for FACS analyses, Figure S7: List of antibodies used for immunofluorescence/immunohistochemistry and FACS.
